# Zinc enhances autophagic flux and lysosomal function through transcription factor EB activation and V-ATPase assembly

**DOI:** 10.3389/fncel.2022.895750

**Published:** 2022-09-29

**Authors:** Ki-Ryeong Kim, Sang Eun Park, Ji-Ye Hong, Jae-Young Koh, Dong-Hyung Cho, Jung Jin Hwang, Yang-Hee Kim

**Affiliations:** ^1^Department of Integrative Bioscience and Biotechnology, Sejong University, Seoul, South Korea; ^2^Asan Institute for Life Sciences, Asan Medical Center, Seoul, South Korea; ^3^Department of Neurology, Asan Medical Center, University of Ulsan College of Medicine, Seoul, South Korea; ^4^Neuronal Injury Lab, Biomedical Research Center, Asan Institute for Life Sciences, Asan Medical Center, Seoul, South Korea; ^5^BK21 FOUR KNU Creative BioResearch Group, School of Life Sciences, Kyungpook National University, Daegu, South Korea

**Keywords:** zinc, lysosome, autophagy, TFEB, cathepsin B, cathepsin D, V-ATPase, neurodegenerative disease

## Abstract

The stimulation of autophagy or lysosomes has been considered therapeutic for neurodegenerative disorders because the accumulation of misfolded proteins is commonly observed in the brains of individuals with these diseases. Although zinc is known to play critical roles in the functions of lysosomes and autophagy, the mechanism behind this regulatory relationship remains unclear. Therefore, in this study, we examined which mechanism is involved in zinc-mediated activation of autophagy and lysosome. Exposure to zinc at a sub-lethal concentration activated autophagy in a concentration-dependent manner in mRFP-GFP-LC3-expressing H4 glioma cells. Zinc also rescued the blocking of autophagic flux arrested by pharmaceutical de-acidification. Co-treatment with zinc attenuated the chloroquine (CQ)-induced increase in the number and size of mRFP-GFP-LC3 puncta in H4 cells and accumulation of p62 by CQ or ammonium chloride in both H4 and mouse cerebrocortical cultures. Zinc rapidly induced the expression of cathepsin B (CTSB) and cathepsin D (CTSD), representative lysosomal proteases in neurons, which appeared likely to be mediated by transcription factor EB (TFEB). We observed the translocation of TFEB from neurite to nucleus and the dephosphorylation of TFEB by zinc. The addition of cycloheximide, a chemical inhibitor of protein synthesis, inhibited the activity of CTSB and CTSD at 8 h after zinc exposure but not at 1 h, indicating that only late lysosomal activation was dependent on the synthesis of CTSB and CTSD proteins. At the very early time point, the activation of cathepsins was mediated by an increased assembly of V-ATPase on lysosomes and resultant lysosomal acidification. Finally, considering that P301L mutation in tau protein causes frontotemporal dementia through aggressive tau accumulation, we investigated whether zinc reduces the accumulation of protein aggregates in SK-N-BE(2)-C neuroblastoma cells expressing wild-type tau or mutant P301L-tau. Zinc markedly attenuated the levels of phosphorylated tau and total tau as well as p62 in both wild-type and mutant tau-overexpressing cells. We also observed that zinc was more effective than rapamycin at inducing TFEB-dependent CTSB and CTSD expression and V-ATPase-dependent lysosomal acidification and CTSB/CTSD activation. These results suggest that the regulation of zinc homeostasis could be a new approach for developing treatments for neurodegenerative diseases, including Alzheimer’s and Parkinson’s.

## Introduction

Autophagy is a self-digestive process that degrades aggregated proteins and damaged organelles to recycle cellular metabolites. Autophagy starts with double-membraned vacuoles, autophagosomes, which surround unnecessary and dysfunctional components, proteins, and intracellular organelles. Autophagosomes fuse with lysosomes to form autolysosomes, the contents of which are degraded by lysosomal acidic hydrolases (Mizushima, 2007; Van Limbergen et al., 2009; Parzych and Klionsky, 2014; Kaur and Debnath, 2015). In particular, autophagy and lysosomal function play a pivotal role in non-dividing neurons for waste clearance. Autophagy/lysosomal function is well-known to be downregulated in neurodegenerative diseases, including Alzheimer’s disease (AD), Parkinson’s disease (PD), Huntington’s disease (HS), and amyotrophic lateral sclerosis (ALS). Failure of autophagy or lysosomal degradation leads to the accumulation of aggregate-prone proteins, such as wild-type or mutant tau and Aβ, mutant α-synuclein, polyglutamine-expanded huntingtin, and mutant TDP-43, in the brains of patients suffering from AD, PD, HD, and ALS, respectively. In accordance with this, loss of Atg genes, for example, *Atg5* and *Atg7*, causes neurodegeneration in mice (Hara et al., 2006; Komatsu et al., 2006). Therefore, the upregulation of autophagy or lysosomal function is considered to have therapeutic effects on neurodegenerative diseases.

Zinc in its ionic form (Zn^2+^) is concentrated in synaptic vesicles in glutamatergic neurons of the central nervous system, which is released upon synaptic activation. The released zinc plays a role as a neurotransmitter by modulating excitatory and inhibitory synaptic transmission extracellularly in the synaptic cleft and intracellularly in postsynaptic neurons (Assaf and Chung, 1984; Howell et al., 1984; Sensi and Jeng, 2004; Sensi et al., 2009). The level of zinc in plasma is also known to decrease with aging, which is more significantly diminished in patients with neurodegenerative disease (Brewer et al., 2010; Vural et al., 2010; Du et al., 2017). In AD-affected brains, zinc is trapped in senile plaques, reducing the intracellular zinc pool and attenuating synaptic activity (Lovell et al., 1998; Grabrucker et al., 2011). In addition, we previously showed that zinc chelation by N,N,N’,N’-tetrakis(2-pyridylmethyl)ethylenediamine (TPEN) induces p53/caspase-dependent neuronal apoptosis in mouse cerebrocortical cultures (Ra et al., 2009), suggesting that severe depletion of zinc induces caspase-dependent neuronal apoptosis (Ahn et al., 1998; Hyun et al., 2001; Zhu et al., 2017). In contrast, under injurious conditions such as ischemia or trauma, the concentration of extracellular zinc rises to hundreds of micromoles and triggers multiple intracellular death pathways leading to necrosis and/or apoptosis (Kim et al., 1999; Hamatake et al., 2000; Truong-Tran et al., 2001; Sensi et al., 2009). Thus, the maintenance of intracellular zinc homeostasis may contribute to overcoming neurodegenerative diseases as well as acute brain injury.

Several studies have reported that zinc plays a role in autophagy and lysosomal functions. Kelleher et al. demonstrated that the vesicular zinc transporter 2 (ZnT2) regulates V-ATPase assembly on lysosomes, driving lysosome biogenesis and acidification (Rivera et al., 2018). Clioquinol, a zinc ionophore, induces autophagy in a zinc-dependent manner and contributes to the clearance of aggregated proteins in astrocytes and neurons (Park et al., 2011). The addition of cilostazol, a phosphodiesterase (PDE) inhibitor, was found to restore autophagic flux blocked by bafilomycin A1 via lysosomal re-acidification in a zinc-dependent manner (Kim et al., 2020). Koh et al. also showed that the H^+^/K^+^-ATPase/ZnT3 complex is recruited to lysosomes in a cAMP-dependent manner and functions as an alternative proton pump for lysosomes when the V-ATPase function is downregulated by bafilomycin A1 (Lee and Koh, 2021). However, under oxidative stress, free zinc in the cytosol is increased, which resulted in the upregulation of zinc level in autophagosomes and lysosomes (Lee and Koh, 2010). Excess zinc leads to lysosomal membrane permeabilization (LMP) and resultant cell death in neurons and glia (Hwang et al., 2008; Lee et al., 2009). These findings collectively suggest that zinc is essential for autophagy and lysosomal functions, but an excess increase in intracellular zinc induces LMP and cell death. Against this background, there is a need to understand the mechanism by which zinc regulates lysosomes and autophagy in order to overcome neurodegenerative diseases.

Lysosomes are essential for eliminating protein aggregates under pathological conditions such as AD, PD, and HD because the final destination of autophagic vacuoles and endocytic vesicles is lysosomes for the digestion of their contents (Kenney and Benarroch, 2015). The primary regulatory process for lysosomal activation is the induction of biogenesis. Transcription factor EB (TFEB) is a major transcription factor for lysosomal biogenesis and autophagy activation. The subcellular localization and activity of TFEB are regulated by mTOR-mediated phosphorylation. Phosphorylated TFEB remains in the cytoplasm, whereas dephosphorylated TFEB translocates to the nucleus to induce the transcription of target genes. TFEB directly binds to coordinated lysosomal expression and regulation (CLEAR) elements, common 10-base E-box-like palindromic sequences, in the promoter region of a large number of genes regulating lysosomal biogenesis, autophagy, and lysosomal exocytosis. TFEB coordinates transcriptional regulation for a cellular degradative pathway. Therefore, to determine whether lysosomal biogenesis is promoted, it is necessary to observe the activation of TFEB.

In this study, we elucidate the precise mechanism by which zinc mediates the activation of lysosomes and autophagy. Because the level of zinc in plasma is significantly reduced in patients with neurodegenerative disease, we examined whether exposure of cortical culture to a sub-lethal concentration of extracellular zinc can activate autophagic flux and lysosomal function, even in the presence of chloroquine, an autophagy inhibitor, and how zinc regulates lysosomal function and autophagy in neurons.

## Materials and methods

### Culture of mouse cerebrocortical neurons, H4, and SK-N-BE(2)-C cells

Mouse cerebrocortical cultures were prepared from embryonic day 13–14 mice. Dissociated cortical cells were plated onto poly-D-lysine (Sigma)-coated plates (SPL), ten hemispheres per six-well plate, in plating medium [Dulbecco’s modified Eagle’s medium (DMEM, GibcoBRL) with 20 mM glucose, 40 mM sodium bicarbonate, 2 mM glutamine, 5% fetal bovine serum (FBS, Hyclone), and 5% horse serum (HS, GibcoBRL) (Kim et al., 1999)]. For near-pure neuronal cultures (PNC), 10 μM cytosine β-D-arabinofuranoside was added to the culture medium at the day in vitro (DIV) 3. All experiments were carried out at DIV 10–11 for mixed cultures, including astrocytes and neurons, and DIV 6–7 for PNC. H4 cells stably expressing mRFP-GFP-LC3 (mRGL-H4 cells) and SK-N-BE(2)-C cells were maintained in the growth medium [DMEM (Welgene) with 4 mM glutamine, 10% FBS (Welgene), 100 units/ml penicillin, and 100 μg/ml streptomycin (Welgene)] at 37°C in a 5% CO_2_ atmosphere.

### Exposure to drugs

Before drug treatment, cultures were washed with serum-free medium [minimum essential medium (MEM, GibcoBRL)]. We used 50 μM chloroquine diphosphate salt (CQ, Sigma), 5 mM ammonium chloride (NH_4_Cl, Sigma), 1 μg/ml actinomycin D (Act.D, Sigma), 10 μg/ml cycloheximide (CHX, Sigma), 500 nM N,N,N’N’-tetrakis(2-pyridylmethyl)ethylenediamine (TPEN), 100–400 nM rapamycin (Sigma), or 2–50 μM ZnCl_2_ (Sigma).

### DNA constructs and transfection

SK-N-BE(2)-C cells were transfected with *GFP-Tau-WT* or *GFP-Tau-P301L* and *RFP-LC3* using Lipofectamine™ 2000 (Thermo Fisher Scientific) and then exposed to drugs after 16 h. *pPK5-EGFP-Tau-WT* and *pPK5-EGFP-Tau-P301L* were obtained from Addgene (#46904 and #46908) and cloned into *pCDNA3.1*, a eukaryotic expression vector. The RFP-LC3 plasmid was a generous gift from Dr. Maria Colombo and Michel Rabinovitch (Universidad Nacional de Cuyo, Mendoza, Argentina), and the mRFP-GFP-LC3 plasmid was a gift from Tamotsu Yoshimori (Addgene plasmid #21074).

### Fluorescence microscopy and confocal imaging

For confocal microscopy, mRGL-H4 cells were cultured on cover glass (circle, 12 mm). After drug treatment, cells were fixed in 4% paraformaldehyde at 4°C for 15 min. The number and size of fluorescent puncta per cell in a microscopic field were measured using the Image J software. In the case of TFEB, PNC was sequentially stained with anti-TFEB antibody (Bethyl, #*A303-673A*), anti-rabbit IgG (H + L) antibody tagged with Alexa Fluor*™* 488 (Invitrogen), and then Hoechst-33258 (Invitrogen) dye. We obtained the fluorescence images using a laser scanning confocal microscope for LC3 puncta (Leica TCS SP5) or a fluorescence microscope (EVOS) for TFEB and Hoechst.

### Western blot assay

Cell lysates were prepared in RIPA lysis buffer (50 mM Tris, pH 7.5, 150 mM NaCl, 1% NP-40, 0.5% Na-Doc, 0.1% SDS, 5 mM EDTA) with freshly prepared protein protease and phosphatase inhibitor (2 μg/ml aprotinin, 1 μg/ml leupeptin, 1 μg/ml pepstatin A, 1 mM phenyl-methylsulfonyl fluoride, 1 mM Na_3_VO_4_, 5 mM NaF, and 10 mM Na_4_P_2_O_7_). Total protein (30–60 μg) was separated by SDS-PAGE (8–15%) under reducing conditions and transferred to a PVDF membrane (INtRON Biotech). Membranes were blocked for 1 h in a blocking buffer containing 3% BSA in TBST buffer [Tris-buffered saline solution and Tween 20 (Duchefa)]. Membranes were immunoblotted with the appropriate antibodies [anti-p62/SQSTM1 (MBL), LC3 (Novus), actin (Sigma), Mucolipin1/TRPML1 (Novus), cathepsin B (Abcam), cathepsin D (Santa Cruz), ATP6V1A (Proteintech), ATP6V1B2 (Abcam), ATP6V0D1 (Proteintech), Lamp-1 (Merck), Cu/Zn-SOD (Stressgen), TFEB (Bethyl, #*A303-673A*), p-Ser/Thr (Abcam), p-tau (Ser396, Upstate), tau (Thermo), or tubulin (Sigma)]. Actin or tubulin was used as a loading control.

### Reverse-transcription polymerase chain reaction

Total RNA was isolated from primary cortical neurons using Trizol reagent (Invitrogen) and reverse-transcribed to cDNA using iScript*™* cDNA synthesis kit (Bio-Rad). The PCR was conducted using the AccuPower PCR Premix Kit (Bioneer) and the following primers and conditions: *ctsb* primers (5′-ATATCACCGGCTTCATGCTTGTATACTCCTGAT-3′ and 5′-TCTGACCGAACCTGCATTCACAAAT-3′) for 33 cycles (94°C for 30 s, 65°C for 1 min, and 72°C for 1 min), *ctsd* primers (5′-ACATCCACTACGGCTCAGGAAGCCT-3′ and 5′-TGCAGCTCCTTCACCTCTTCCACA-3′) for 35 cycles (94°C for 30 s, 61°C for 1 min, 72°C for 30 s), *trpml1 primers* (5′-GTCGGTGTCATTCGCTACCTGA-3′ and 5′-GAACGATCCAGCCACAGAAGCA-3′) for 33 cycles (94°C for 60 s, 63°C for 1 min, and 72°C for 1 min), and *β-actin* primers (5′-TCTACAAATGTGGCTGAGGAC-3′ and 5′-CCTGGGCCATTCAGAAATTA-3′) for 33 cycles (94°C for 30 s, 65°C for 1 min, and 72°C for 1 min). PCR products were electrophoresed on a 2% agarose gel, stained with ethidium bromide, and then visualized using BIS 303PC (DNR Bio-imaging System Ltd.).

### *In situ* cathepsin enzymatic activity assay

In situ cathepsin B activity assay was performed using the Magic Red Cathepsin B Detection Kit (Immunochemistry) in accordance with the manufacturer’s protocol. After drug treatment, cells were exposed to Magic Red Staining Solution 1× for 1 h at 37*°C* in the dark. In situ Cathepsin D activity was measured by pepstatin A, Bodipy^®^ FL Conjugate (Thermo Fisher *Scientific*). Drug-treated cells were loaded with a final concentration of 1 μ M Bodipy-FL-pepstatin A for 1 h at 37*°C* in the dark. Stained cells were fixed with 4% paraformaldehyde and washed with DPBS. Samples were examined under a fluorescence microscope (EVOS). The mean intensity of fluorescence was quantified in a given microscopic field using Image J software.

### Immunoprecipitation

Cell lysis was performed using RIPA lysis buffer with freshly prepared protein protease and phosphatase inhibitor. For the immunoprecipitation, more than two volumes of PBS were added to cell lysates, and 100 μg of protein was used for each sample. One microgram of anti-TFEB antibody (Bethyl, #*A303-673A*) and 30 μl of Protein A/G PLUS-Agarose beads (Santa Cruz) were used. Immunoblotting was performed with the appropriate antibodies [p-Ser/Thr (Abcam), TFEB (Bethyl, #*A303-673A*), or actin (Sigma)] and chemiluminescence signals were detected by MF-chemibis3.2 (DNR Bio-imaging System Ltd.).

### Lysosomal pH measurement and live cell imaging

Quantification of lysosomal pH was performed using a ratiometric lysosomal pH dye, Lysosensor Yellow/Blue DND-160 (Invitrogen). In brief, the mouse cerebrocortical cultures were labeled with 1 μM Lysosensor Yellow/Blue DND-160 for 30 min at 37°C in MEM and then excessive dye was washed out with MEM. The labeled cells were treated with drugs or zinc for the indicated times. To make the pH calibration curve, the labeled cultures were exposed to standard solutions [20 μM monensin (Enzo), 10 μM nigericin (Enzo), 5 mM NaCl (Sigma), 110 mM KCl (Sigma) in 20 mM 2-(N-morpholino) ethanesulfonic acid (MES, Sigma) buffer, pH 3.0–6.0] for 10 min. Dual fluorescence emitted at 440 and 535 nm in response to excitation at 340 and 380 nm was measured using a microplate reader (Gemini EM, Molecular Devices). Following a previous report (Lu et al., 2014), the measured fluorescence ratio (em 440/535) was substituted into a standard curve. The obtained value was plotted as a pH value on a graph. Live confocal images of H4 cells labeled with LysoTracker Red DND-99 (Invitrogen, ex 580 nm and em 615 nm) were obtained using an LSM880 Confocal Live-Cell Imaging System (Carl Zeiss, Oberkochen, Germany).

### Fractionation of protein extracts

Mouse cerebrocortical cultures were harvested in homogenization buffer [250 mM sucrose, 3 mM imidazole (pH 8.0), 0.5 mM EDTA, and freshly prepared protein protease and phosphatase inhibitors], and then homogenized at 4°C by seven passages through a 23G needle fitted on a 1 ml syringe. One-fifth of the cell lysates were used for protein expression, and the remainder was further centrifuged at 3,000 rpm (750 × g) and 4°C for 10 min. The supernatants were collected as post-nuclear supernatants. Post-nuclear supernatant samples were centrifuged at 110,000 × g and 4°C for 1 h. The supernatants were used as the cytosolic fraction, and the pellets were used as the membrane fraction.

### Statistical analysis

All statistical comparisons were performed by a two-tailed Student’s t-test for single comparisons or one-way ANOVA with an appropriate *post hoc* test for multiple comparisons as indicated in the figure legend. A *P* value less than 0.05 was considered statistically significant.

## Results

### Zinc activates autophagy

**FIGURE 1:**
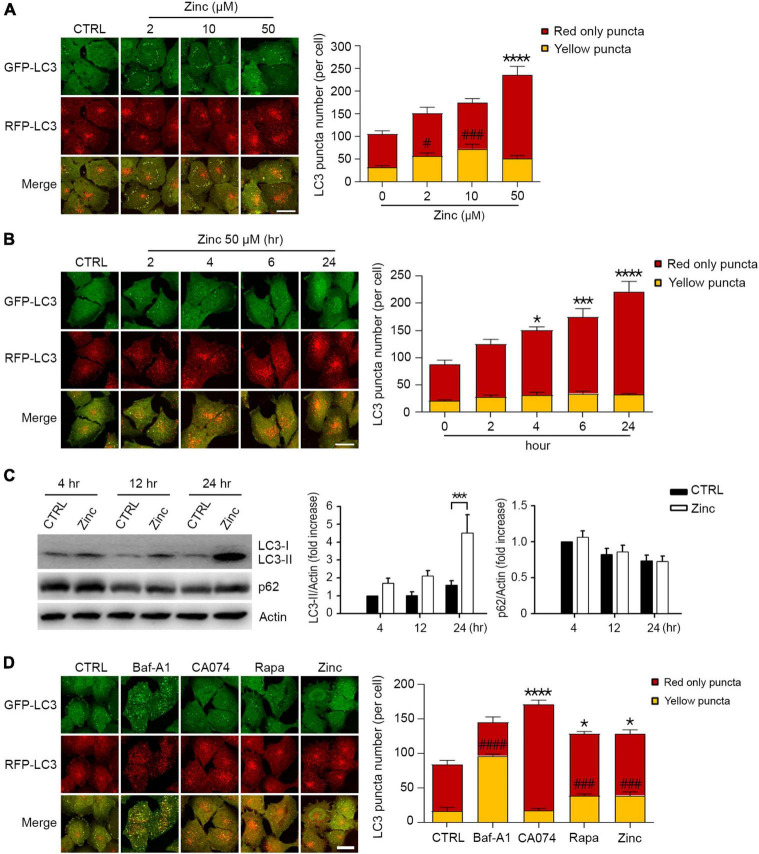
Zinc activates autophagic flux. **(A)** Fluorescence confocal microscopy images of H4 glioma cells expressing mRFP-GFP-LC3 (mRGL-H4 cells) 24 h after exposure to the indicated concentrations of ZnCl_2_ (Zinc). The bar graph (right) represents the quantitative analysis of the number of red-only or yellow puncta per cell (mean ± SEM, *n* = 17 different fields taken from ≥3 independent biological replicate experiments). Scale bar 20 μm. ****p < 0.0001 for red-only puncta, and ^#^*p* < 0.05 or ^###^*p* < 0.001 for yellow puncta by ANOVA with *post hoc* Dunnett’s test. **(B)** Fluorescence confocal microscopy images of mRGL-H4 cells at the indicated time points after exposure to 50 μM ZnCl_2_ (Zinc). The bar graph (right) represents the quantitative analysis of the number of red-only or yellow puncta per cell (mean ± SEM, *n* = 10 different fields taken from ≥3 independent biological replicate experiments). Scale bar 20 μm. *p < 0.05, ***p < 0.001, or ****p < 0.0001 for red-only puncta by ANOVA with *post hoc* Dunnett’s test. **(C)** Western blot analysis for LC3 and p62 in mRGL-H4 cells. Protein samples were prepared at the indicated time points after exposure to sham wash (CTRL) or 50 μM ZnCl_2_ (Zinc). Actin was used as the loading control. Quantification of LC3-II conversion and p62 expression at the indicated time points was made in ≥3 independent biological replicate experiments. ***p < 0.001 by ANOVA with *post hoc* Dunnett’s test. **(D)** Fluorescence confocal microscopy images of mRGL-H4 cells 12 h after sham wash (CTRL), or exposure to 100 nM bafilomycin A1 (Baf-A1), 10 μM CA074-ME (CA074), 100 nM rapamycin (Rapa), or 50 μM ZnCl_2_ (Zinc). Quantification of the number of red-only or yellow puncta per cell (mean ± SEM, *n* = 15 different fields taken from ≥3 independent biological replicate experiments) was shown as right bar graph. Scale bar 20 μm. *p < 0.05 or ****p < 0.0001 for red-only puncta, and ^###^*p* < 0.001 or ^####^*p* < 0.0001 for yellow puncta by ANOVA with *post hoc* Dunnett’s test.

Since several reports have suggested the critical role of zinc in lysosomal function and autophagy (Park et al., 2011; Liuzzi et al., 2014; Koh et al., 2019), we examined whether the exposure of H4 neuroglioma cells stably overexpressing mRFP-GFP-tagged LC3 (mRGL-H4 cells) to zinc in culture medium activated autophagic flux. A sub-lethal concentration of zinc increased the number of autophagic vesicles (AVs), including autophagosomes (APs) and autolysosomes (ALs), in a concentration-dependent manner in mRGL-H4 cells (Figure 1A). Since the GFP signal is easily quenched in acidic compartments and RFP fluorescence is more stable (Kimura et al., 2007), red-only puncta represent ALs, and both RFP- and GFP-positive yellow puncta represent APs. Zinc significantly augmented ALs rather than APs from 2 h after treatment with 50 μM zinc, continuously increasing until 24 h (Figure 1B), suggesting the possibility that the increase in AVs caused by zinc likely results from the activation rather than inhibition of autophagy. Furthermore, since significant rise in endogenous LC3-II and no accumulation of p62 was observed after zinc treatment (Figure 1C), the increase in AVs may be due to the activation of autophagy. To confirm whether zinc-increased AVs come from autophagy activation, we compared the numbers of APs and ALs after exposure to well-known autophagy- or lysosome-regulating agents, bafilomycin A1 (Baf-A1), CA074-ME (CA074), or rapamycin. Baf-A1, which causes lysosomal alkalization and inhibits the fusion of APs with lysosomes, dramatically increased yellow puncta compared with the level in control (Figure 1D). CA074, a specific inhibitor of cathepsin B (CTSB), does not block the fusion of APs and lysosomes and inhibits lysosomal degradation, thereby accumulating ALs, red-only puncta (Figure 1D). Rapamycin, an autophagy activator, increased both yellow and red-only puncta like zinc (Figure 1D), indicating that zinc may activate autophagy.

### Zinc restores autophagic flux suppressed by an inhibitor of autophagy

**FIGURE 2:**
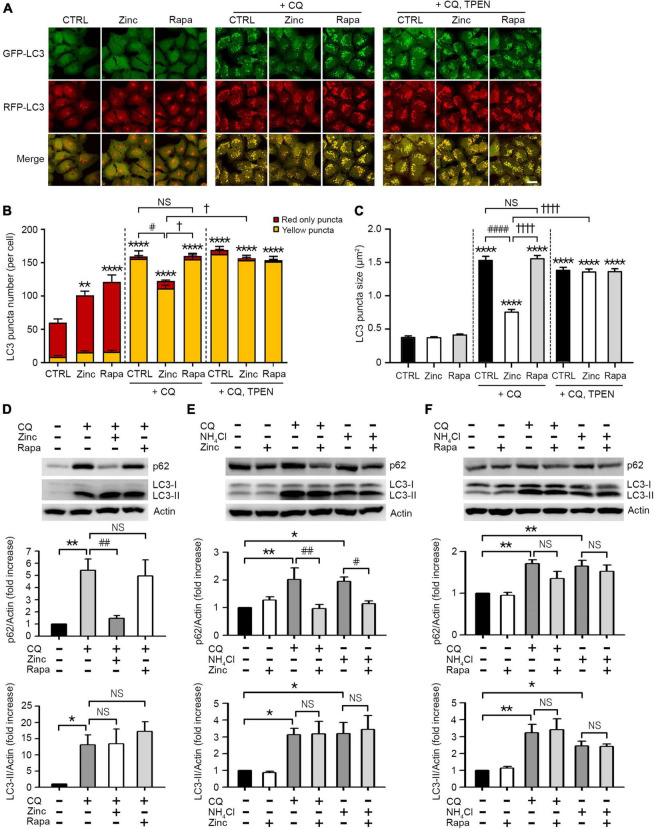
Zinc rescues the blockade of autophagic flux. **(A)** Fluorescence confocal microscopy images of mRGL-H4 cells 12 h after sham wash (CTRL) or exposure to 50 μM ZnCl_2_ (Zinc) or 100 nM rapamycin (Rapa) in the presence or absence of 50 μM chloroquine (CQ) or CQ plus TPEN (+CQ, TPEN). Scale bar 25 μm. **(B,C)** Quantitative analysis of RFP-GFP-LC3 puncta observed 12 h after exposure to zinc or rapamycin (Rapa) in the presence or absence of chloroquine (CQ) or CQ plus TEPN (+CQ, TPEN). The bar graphs represent the total number of RFP-GFP-LC3 puncta including red-only and yellow puncta per cell **(B)** and the mean size of red-only and yellow puncta (C) (mean ± SEM, *n* = 12 different fields taken from ≥3 independent biological replicate experiments). **p < 0.01 or ****p < 0.0001 vs. sham wash CTRL, ^#^*p* < 0.05 or ^####^*p* < 0.0001 vs. CQ alone, and ^†^*p* < 0.05 or ^††††^*p* < 0.0001 vs. CQ + zinc by ANOVA with *post hoc* Dunnett’s test. There was no significant difference between CQ alone and CQ plus rapamycin (NS). **(D)** Western blot analysis for p62 and LC3 in mRGL-H4 cells. Protein samples were prepared at 9 h after exposure to sham wash (CTRL), or 50 μM chloroquine with or without 50 μM ZnCl_2_ (Zinc) or 100 nM rapamycin (Rapa). Quantification of the expression level of p62 and LC3-II was performed in ≥ 3 independent biological replicate experiments. *p < 0.05 or **p < 0.01 vs. sham wash CTRL, and ^##^*p* < 0.01 vs. CQ alone by ANOVA with *post hoc* Dunnett’s test. There was no significant difference between CQ alone and CQ plus rapamycin (NS). **(E,F)** Western blot analysis for p62 and LC3 in mouse cerebrocortical cultures. Protein samples were prepared at 12 h after exposure to sham wash (CTRL), 50 μM chloroquine (CQ), or 5 mM ammonium chloride (NH_4_Cl) in the presence or absence of 25 μM ZnCl_2_ (Zinc, E) or 100 nM rapamycin (Rapa, F). Quantification of the expression level of p62 and LC3-II was performed in ≥3 independent biological replicate experiments. *p < 0.05 or **p < 0.01 vs. CTRL, and ^#^*p* < 0.05 or ^##^*p* < 0.01 vs. CQ or NH_4_Cl alone by ANOVA with *post hoc* Dunnett’s test. There was no significant difference between CQ or NH4Cl alone and CQ or NH4Cl plus rapamycin (NS).

In addition to autophagy activation by zinc, we investigated whether zinc can rescue the blockade of autophagic flux. To arrest autophagic flux, we used chloroquine (CQ) in mRGL-H4 cells, which blocks the fusion of autophagosomes with lysosomes by de-acidification of lysosomes. CQ dramatically accumulated APs appeared as yellow puncta in mRGL-H4 cells (Figures 2A,B) and markedly increased the size of LC3 puncta, too (Figures 2A,C). Interestingly, co-treatment with zinc notably reduced CQ-increased LC3 puncta in both number and size (Figures 2A–C), but rapamycin did not produce a significant change (Figures 2A–C). Since co-exposure to zinc and TPEN reversed the effect of zinc on CQ-induced LC3 puncta accumulation (Figures 2A–C), we confirmed that the rescue from autophagy blockade was a zinc ion-specific reaction. Consistent with this, we observed that CQ accumulated a massive amount of LC3-II and p62 protein in Western blot (Figure 2D). While the increase of LC3-II by CQ was not affected by zinc or rapamycin, the p62 protein level was reversed by co-treatment with zinc but not by rapamycin (Figure 2D). We confirmed these results in mouse primary cortical neuronal cultures. Exposure to zinc significantly diminished the accumulation of p62 protein by CQ or ammonium sulfate (NH_4_Cl) (Figure 2E). However, rapamycin did not significantly attenuate p62 accumulation (Figure 2F). These results suggested that rapamycin is insufficient to rescue the blocking of autophagic flux by CQ or NH_4_Cl in H4 or mouse cerebrocortical neuronal cultures, although it is a potent activator of autophagy. However, zinc alleviated the suppression of autophagic flux as well as activating autophagy in both neuroglioma H4 cells and primary neuronal cultures.

### Zinc induces the expression of cathepsin B and D via TFEB activation

**FIGURE 3:**
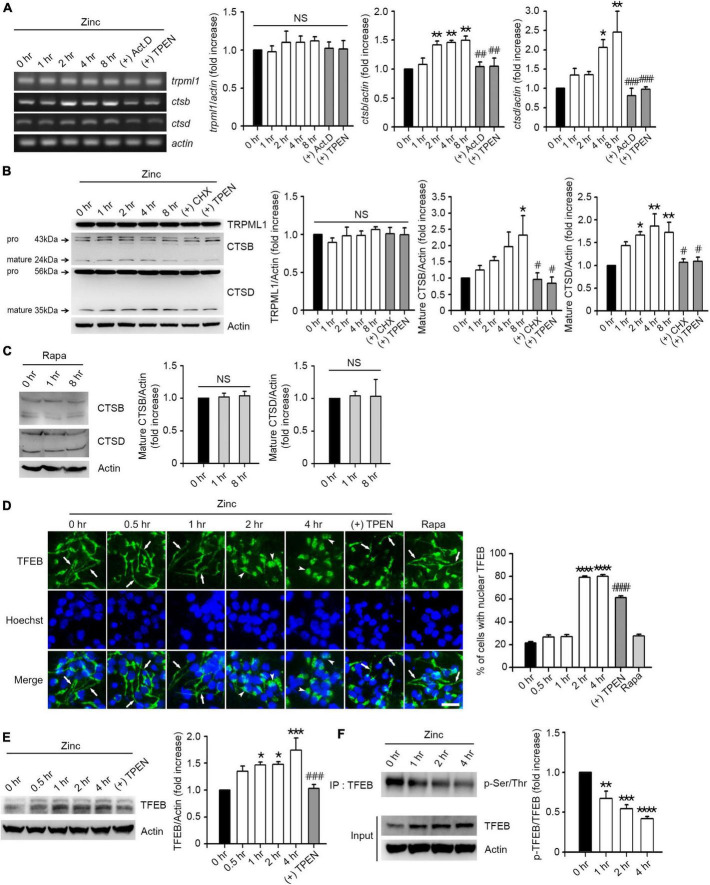
Zinc induces *de novo* synthesis of CTSB and CTSD via TFEB activation. **(A)** Reverse-transcription polymerase chain reaction (RT-PCR) analysis for *trpml1*, *ctsb*, and *ctsd* in mouse cerebrocortical cultures. mRNA samples were prepared at the indicated time points after exposure to 25 μM ZnCl_2_ (Zinc) with or without 1 μg/ml actinomycin D (+Act.D) or 500 nM TPEN (+TPEN). Quantification of mRNA levels of *trpml1*, *ctsb*, and *ctsd* was performed in ≥3 independent biological replicate experiments. *p < 0.05 or **p < 0.01 vs. CTRL (0 h), and ^##^*p* < 0.01 or ^###^*p* < 0.001 vs. zinc (8 h) by ANOVA with *post hoc* Dunnett’s test. There was no significant difference in trpml1 mRNA levels (NS). **(B,C)** Western blot analysis for TRPML1, CTSB and CTSD in mouse cerebrocortical cultures. Proteins were prepared at the indicated time points after exposure to 25 μM ZnCl_2_ (Zinc) with or without 10 μg/ml cycloheximide (+CHX) or 500 nM TPEN (+TPEN) **(B)**, or 100 nM rapamycin (Rapa) **(C)**. Quantification of TRPML1 and mature forms of CTSB and CTSD was performed in ≥3 independent replicate experiments. *p < 0.05 or **p < 0.01 vs. CTRL (0 h), and ^#^*p* < 0.05 vs. zinc (8 h) by ANOVA with *post hoc* Dunnett’s test. There was no significant difference between sham wash control and rapamycin (NS). **(D)** TFEB- and Hoechst-stained confocal microscope images of mouse near-pure cortical neuronal culture at the indicated time points after exposure to 25 μM ZnCl_2_ (Zinc) with or without 500 nM TPEN (+TPEN) or at 4 h after exposure to 100 nM rapamycin (Rapa). Arrows indicate TFEB in neurites, and arrowheads indicate TFEB in the nucleus. Scale bar, 25 μm. The bar graph (right) represents the quantitative analysis of the number of cells to show TFEB translocation into the nucleus (mean ± SEM, *n* = 15 different fields taken from ≥3 independent biological replicate experiments). ****p < 0.0001 vs. CTRL (0 h), and ^####^*p* < 0.0001 vs. zinc (4 h) by ANOVA with *post hoc* Dunnett’s test. **(E)** Western blot analysis for TFEB in mouse cerebrocortical cultures. Protein samples were prepared at the indicated time points after exposure to 25 μM ZnCl_2_ (Zinc) with or without 500 nM TPEN (+ TPEN). *p < 0.05 or ***p < 0.001 vs. CTRL (0 h), and ^###^*p* < 0.001 vs. zinc (4 h) by ANOVA with *post hoc* Dunnett’s test. **(F)** Western blot analysis for TFEB phosphorylation in mouse cerebrocortical cultures. Protein extracts were immunoprecipitated with anti-TFEB antibody and then immunoblotted using anti-pSer/Thr antibody. Quantification of the ratio of p-TFEB to TFEB was performed in ≥3 independent replicate experiments. **p < 0.01, ***p < 0.001, or **** *p* < 0.0001 by ANOVA with *post hoc* Dunnett’s test.

Since zinc resolved the blocked autophagic flux, we hypothesized that zinc also affects lysosomal function. First, we examined whether zinc changed the expression of transient receptor potential mucolipin 1 (TRPML1), a lysosomal calcium/zinc channel, or cathepsin B (CTSB) and D (CTSD), the major lysosomal proteases in neurons. Although TRPML1 was not affected by zinc, the expression level of CTSB and CTSD was induced by zinc (Figures 3A,B). Treatment with zinc significantly increased the mRNA levels of *ctsb* from 2 to 8 h and *ctsd* from 4 to 8 h (Figure 3A). The increase in mRNA of *ctsb* and *ctsd* was reversed by the addition of actinomycin D (Act.D), an RNA synthesis inhibitor, or TPEN at 8 h (Figure 3A). In the protein levels of CTSB and CTSD, only mature forms of them were increased by zinc in a *de novo* synthesis-dependent manner (Figure 3B). The addition of a protein synthesis inhibitor, cycloheximide (CHX), or TPEN, reversed the increase in their protein levels induced by 8 h after zinc treatment (Figure 3B). However, rapamycin did not increase the CTSB or CTSD expression in mouse cerebrocortical cultures (Figure 3C), suggesting that zinc but not rapamycin induces the *de novo* synthesis of CTSB and CTSD.

Next, we examined whether zinc activates TFEB, a master transcription factor for lysosomal biogenesis, to induce an increase in CTSB/CTSD expression. We observed that TFEB resided in both soma and neurites in mouse cerebrocortical cultures (Figure 3D). From 2 h after zinc treatment, TFEB in neurites dramatically translocated to soma including the nucleus (Figure 3D), suggesting the activation of TFEB. Zinc-induced nuclear translocation of TFEB was reversed by TPEN, a zinc chelator (Figure 3D). Consistent with the lack of induction of CTSB/CTSD by rapamycin, TFEB translocation to the nucleus after rapamycin treatment was not detected (Figure 3D). Moreover, the amount of TFEB protein was increased by zinc from 1 h after zinc treatment (Figure 3E). Since the dephosphorylation of TFEB is needed for the nuclear translocation and activation of TFEB, we investigated whether zinc decreased the phosphorylation levels of TFEB. From 1 h after zinc treatment, we detected the reduction of phosphorylation levels of TFEB (Figure 3F), indicating the activation of TFEB.

### Zinc promotes rapid activation of cathepsin B and D

**FIGURE 4:**
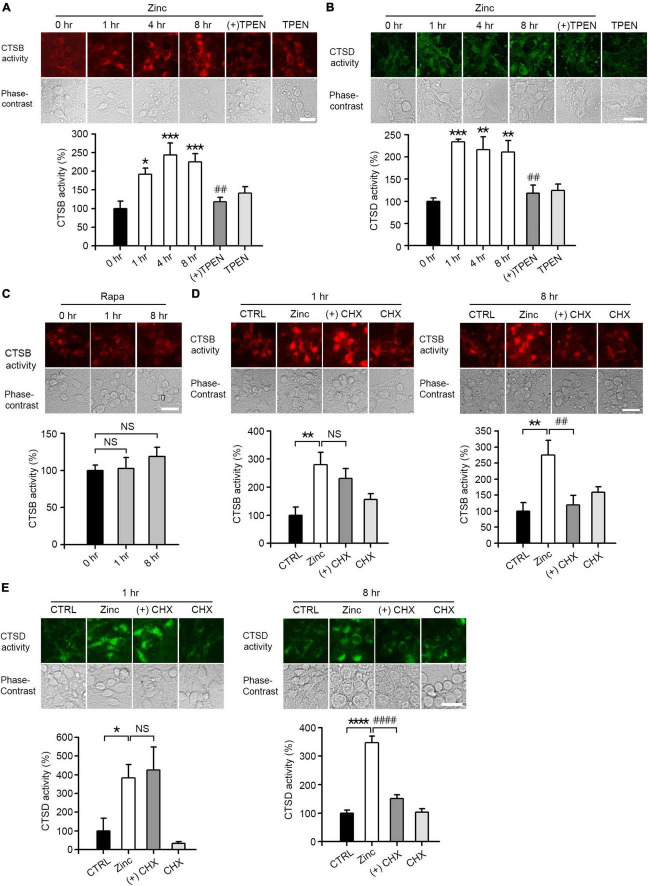
Zinc increases CTSB and CTSD activity. **(A,B)** *In situ* enzyme activity analysis for CTSB **(A)** and CTSD **(B)** in mouse near-pure cerebrocortical neuronal cultures. The fluorescence signal of the cleaved substrates of CTSB or CTSD was observed at the indicated time points after exposure to 25 μM ZnCl_2_ (Zinc) with or without 500 nM TPEN (+TPEN) or TPEN alone. The bar graph represents quantitative analysis of the fluorescence intensity (mean ± SEM, *n* ≥ 5 different fields taken from ≥4 independent biological replicate experiments). Scale bar: 25 μm, *p < 0.05, **p < 0.01 or ***p < 0.001, and ^##^*p* < 0.01 vs. zinc (8 h) by ANOVA with *post hoc* Dunnett’s test. **(C)** *In situ* enzyme activity analysis for CTSB in mouse near-pure cerebrocortical neuronal cultures. The fluorescence signal of the cleaved substrates of CTSB was observed at the indicated time point after exposure to 100 nM rapamycin (Rapa). The bar graph represents quantitative analysis of the fluorescence intensity of the cleaved substrates of CTSB (mean ± SEM, *n* ≥ 6 different fields taken from ≥ 3 independent biological replicate experiments). There was no significant difference between sham wash control and rapamycin (NS). Scale bar: 25 μm. **(D,E)** *In situ* enzyme activity analysis for CTSB **(D)** and CTSD **(E)** in mouse near-pure cerebrocortical neuronal cultures. CTSB or CTSD activity in lysosomes was observed at 1 or 8 h after exposure to sham wash (CTRL), 25 μM ZnCl_2_ alone (Zinc), zinc plus 10 μg/ml cycloheximide (+CHX), or CHX alone. The bar graphs represent quantitative analysis of the fluorescence intensity of the cleaved substrates of CTSB **(D)** or CTSD **(E)** (mean ± SEM, *n* = 7 different fields taken from ≥3 independent biological replicate experiments). Scale bar: 25 μm, *p < 0.05, **p < 0.01 or ****p < 0.0001 vs. sham wash control and ^##^*p* < 0.01 or ^####^*p* < 0.0001 vs. zinc (8 h) by ANOVA with *post hoc* Dunnett’s test. There was no significant difference between zinc alone and zinc plus cycloheximide (NS).

We performed *in situ* enzyme assay to determine whether treatment with zinc led to substantial changes in enzymatic activity of CTSB and CTSD in cortical neuronal cells. The activities of CTSB and CTSD were increased about two times after 1 h of zinc treatment and maintained until 8 h (Figures 4A,B). Unlike zinc, rapamycin had little effect on the activity of CTSB (Figure 4C). Since we observed that zinc significantly increased the enzymatic activity of CTSB and CTSD from 1 h when the expression levels of CTSB/CTSD were unchanged, we assumed that the increase in enzyme activity of CTSB and CTSD at early times might be independent of their *de novo* synthesis. To confirm this possibility, *in situ* cathepsin activity assay was performed at 1 and 8 h after zinc treatment in the presence or absence of CHX, a protein synthesis inhibitor. One hour after zinc treatment, CHX did not change the activation of CTSB by zinc (Figure 4D). On the other hand, at 8 h, CHX decreased the activation of CTSB by zinc (Figure 4D). Similar to CTSB, the addition of CHX did not affect the activity of CTSD increased by zinc at 1 h but decreased it at 8 h (Figure 4E). These findings suggest that the rapid activation of CTSB and CTSD by zinc at 1 h is independent of protein synthesis.

### Zinc very quickly acidifies lysosomal pH via assembly of V-ATPase

**FIGURE 5:**
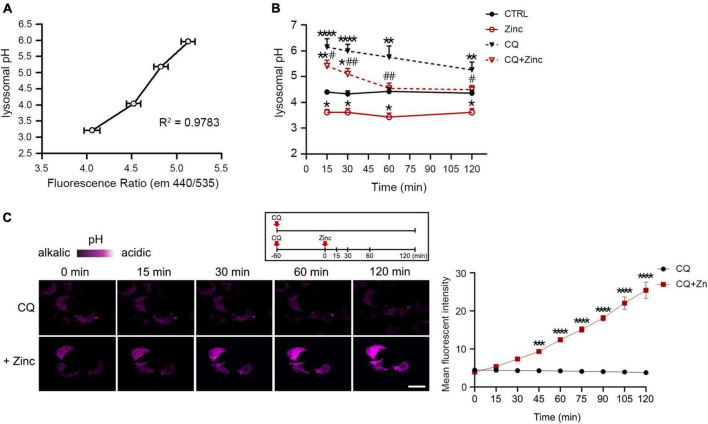
Zinc rapidly acidifies lysosomal pH. **(A,B)** Measurement of intra-organellar pH in cerebrocortical neuronal cultures using the ratiometric dye LysoSensor™ Yellow/Blue DND-160. Emission was obtained at 440 and 535 nm for excitation at 340 and 380 nm, respectively. A graph was generated based on the fluorescence intensity ratio (440/535 nm). For the pH calibration curve **(A)**, pH 3.21, 4.03, 5.18, and 5.96 solutions were used. Lysosomal pH of cortical cultures (**B**) was detected at the indicated time points after exposure to sham wash (CTRL), 25 μM ZnCl_2_, 50 μM chloroquine, or CQ plus Zinc (mean ± SEM, *n* ≥ 8 from ≥ 4 independent biological replicate experiments). *p < 0.05, **p < 0.01 or ****p < 0.0001 vs. sham wash control and ^#^*p* < 0.05 or ^##^*p* < 0.01 vs. CQ alone by ANOVA with *post hoc* Dunnett’s test. **(C)** Live-cell confocal microscopy images of H4 cells stained with LysoTracker Red DND-99. Cells were pretreated with 50 μM chloroquine (CQ) for 1 h, and then the live images were taken with or without 50 μM ZnCl_2_ for an additional 2 h. Scale bar, 20 μm. The graph represents the mean fluorescent intensity of pseudo-color quantified in 20 cells. ***p < 0.001 or ****p < 0.0001 by ANOVA with *post hoc* Bonferroni’s test.

Because cathepsins in the lysosomes can be activated when the lysosomal pH is lowered (Turk et al., 1995, 1999; Yadati et al., 2020), zinc-induced cathepsin activation at 1 h might be the result of lysosomal acidification. Therefore, we examined whether lysosomal pH can be regulated by zinc. The measurement of intraorganellar pH was performed using the LysoSensor™ Yellow/Blue DND-160 dyes. We presented pH calibration curves in Figure 5A and corresponding results of mouse cerebrocortical cultures after exposure to zinc, CQ, or CQ plus zinc in Figure 5B. Treatment with zinc started to acidify lysosomes from 15 min compared with the pH in the control, which was maintained until 2 h (Figure 5B). Moreover, a rise of lysosomal pH by CQ was also lowered by the addition of zinc from 15 min (Figure 5B). At 1 h after exposure to CQ and zinc, CQ-induced alkalization was revered to basal control levels (Figure 5B). Changes in lysosomal acidity in H4 cells were also detected through live images using LysoTracker, a probe for acidic organelles (Figure 5C). Treatment with CQ for 1 h dramatically reduced the fluorescent intensity, which was maintained up to 2 h. On the contrary, the addition of zinc markedly recovered the fluorescent intensity lowered by CQ exposure. This means that lysosomal pH can be rapidly re-acidified by zinc.

**FIGURE 6:**
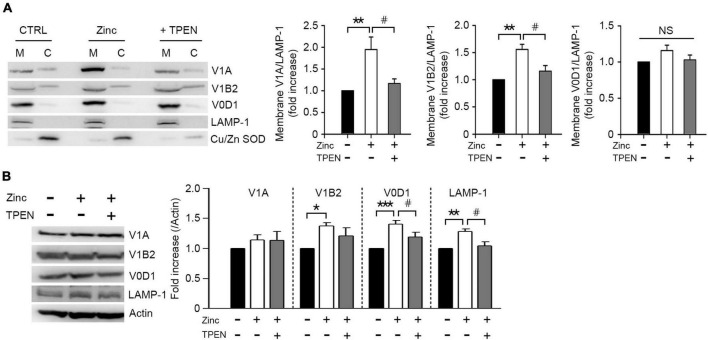
Zinc promotes vacuolar-type H^+^–ATPase assembly. Western blot analysis for the membrane and cytosolic distribution **(A)** and expression **(B)** of V1 and V0 subunits of V-ATPase in mouse cerebrocortical cultures. Protein samples were prepared at 1 h after exposure to sham wash (CTRL) or 25 μM ZnCl_2_ (Zinc) with or without TPEN. Total protein extracts were used for expression level **(B)** and then separated into membrane (M) and cytosolic (C) fractions **(A)**. Antibodies against V1A or V1B2 were used for V1 subunit, and V0D1 was used for V0 subunit of V-ATPase. LAMP-1 and Cu/Zn-SOD were used for verification of the quality of membrane and cytosolic separation. Bar graphs **(A)** represent the ratio of V1A, V1B2, or V0D1 to LAMP-1 level in the membrane fraction (mean ± SEM, taken from ≥ 4 independent experiments). **p < 0.01 vs. sham wash control and ^#^*p* < 0.05 vs. zinc alone by ANOVA with *post hoc* Dunnett’s test. There was no significant difference in V0D1 levels on the membrane (NS). Expression levels of V1A, V1B2, V0D1, and LAMP-1 **(B)**. Quantification was made in ≥3 independent biological replicate experiments. *p < 0.05, **p < 0.01, or ***p < 0.001 vs. sham wash control and ^#^*p* < 0.05 vs. zinc alone by ANOVA with *post hoc* Dunnett’s test.

Lysosomal acidification was mainly mediated by vacuolar H^+^-ATPase (V-ATPase), which is activated by a reversible assembly of cytoplasmic V1 domain with membrane-bound V0 domain (McGuire et al., 2017). To elucidate whether zinc would induce V-ATPase assembly, we analyzed the level of V1A or V1B2, one of the V1 subunits bound to the membrane. Zinc significantly increased V1A and V1B2 associated with membrane in cortical neuronal cultures at 1 h (Figure 6A). We next investigated whether the expression of V1 or V0 subunits was affected by zinc considering that V-ATPase expression can be enhanced by TFEB activation (Pena-Llopis et al., 2011). At 1 h after exposure to zinc in cortical cultures, the expression levels of V1B2, V0D1, and Lysosomal-associated membrane protein 1 (LAMP-1) were significantly increased (Figure 6B). Taking these findings together, we suggested that zinc induces the recruitment of V1A domain into the membrane and an increase in the expression of V1/V0 subunits to make the active form of V-ATPase, which facilitates lysosomal acidification.

### Zinc diminishes the accumulation of tau protein

**FIGURE 7:**
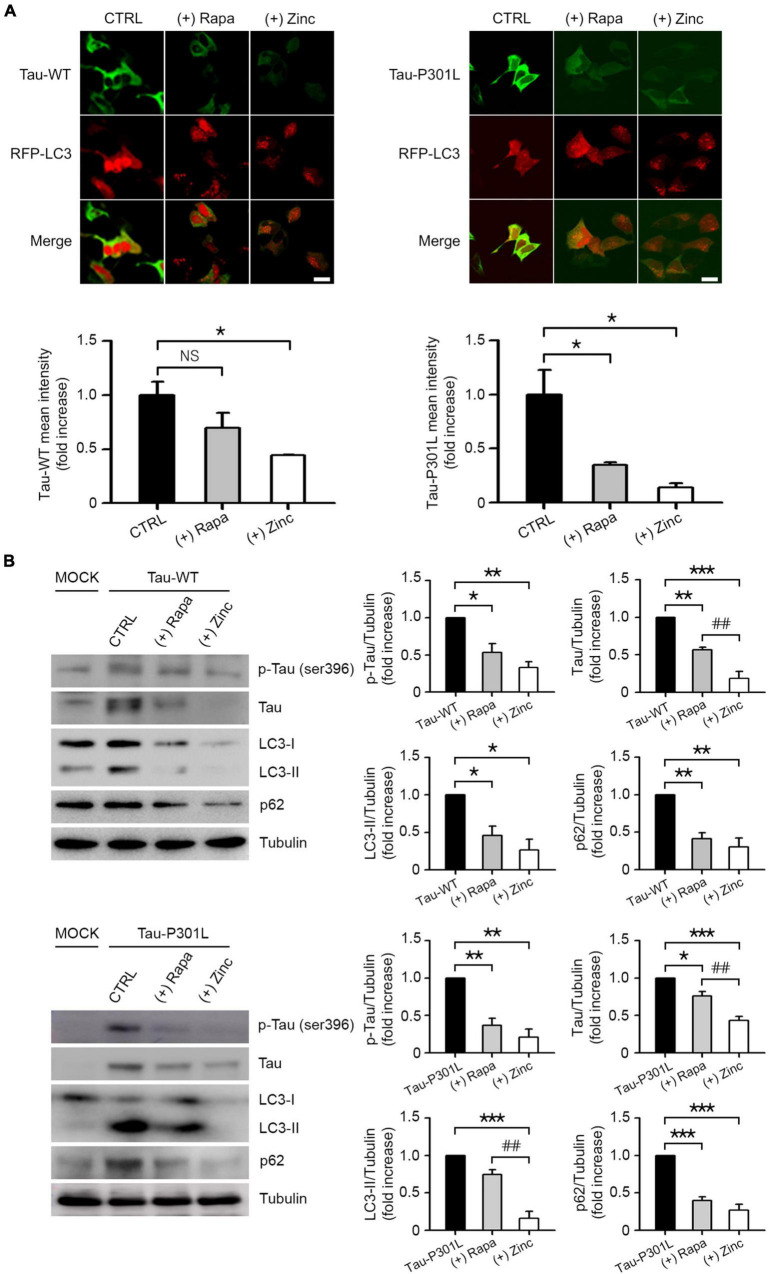
Zinc-activated lysosomal function and autophagic flux contribute to the degradation of wild-type tau or mutant tau(P301L). **(A)** SK-N-BE(2)-C neuroblastoma cells expressing RFP-LC3 were transiently transfected with GFP-Tau-WT (Tau-WT) or GFP-Tau-P301L (Tau-P301L). Fluorescence confocal microscopy images of SK-N-BE(2)-C cells at 6 h after sham wash (CTRL), or exposure to 400 nM rapamycin (+Rapa) or 30 μM ZnCl_2_ (+Zinc). The bar graph represents the fluorescence intensity of GFP-Tau-WT or GFP-Tau-P301L (mean ± SEM, taken from ≥3 independent biological replicate experiments). Scale bar: 10 μm. *p < 0.05 by ANOVA with *post hoc* Bonferroni’s test. **(B)** Western blot analysis for phospho-tau at Ser396, tau, LC3, and p62 in SK-N-BE(2)-C neuroblastoma cells transiently expressing GFP-Tau-WT (Tau-WT) or GFP-Tau-P301L (Tau-P301L). Protein samples were prepared at 6 h after exposure to sham wash (CTRL), 400 nM rapamycin (+Rapa), or 30 μM ZnCl_2_ (+Zinc). Tubulin was used as a loading control. Quantification of protein levels of p-tau, tau, LC3-II, and p62 was performed in ≥3 independent biological replicate experiments. *p < 0.05, **p < 0.01, ***p < 0.001 or ****p < 0.0001 vs. control and ^##^*p* < 0.01 vs. rapamycin by ANOVA with *post hoc* Bonferroni’s test.

In several neurodegenerative diseases, including AD and FTD, it has been reported that deficiency of autophagy or lysosomal function leads to the accumulation of aggregate-prone proteins such as mutant or phosphorylated tau or Aβ (Hamano et al., 2008; Haass et al., 2012; Xin et al., 2018). Therefore, we analyzed the contribution of zinc-induced autophagic flux or lysosomal activation to the clearance of mutant tau proteins. When we transfected GFP-fused wild-type Tau (Tau-WT) or GFP-fused P301L mutant tau (Tau-P301L) into RFP-LC3-overexpressing SK-N-BE(2)-C neuroblastoma cells, we observed accumulations of tau and large LC3 puncta in the perinuclear region, and changes in cell morphology as cells shrank and detached from the dish (Figure 7A). These phenomena were more severe in Tau-P301L-expressing cells than in Tau-WT cells. Overexpression of tau proteins dramatically increased phosphorylation of tau on serine 396 and led to autophagic arrest, which was represented by the accumulation of LC3-II and p62 (Figure 7B). The addition of rapamycin or zinc notably reduced tau accumulation and phosphorylation, as well as LC3-II and p62 accumulation by Tau-WT or Tau-P301L overexpression. In all cases, the reduction by zinc was greater than that by rapamycin (Figures 7A,B). The effects of zinc on the degradation and dephosphorylation of tau, and the rescue from autophagic flux arrest were much more pronounced than those of rapamycin.

## Discussion

**FIGURE 8:**
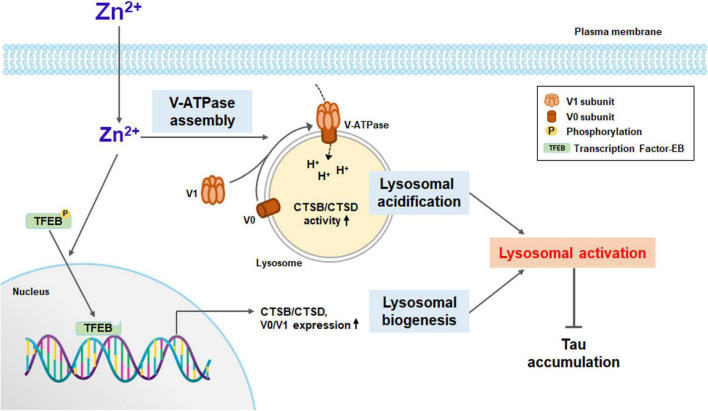
A diagram for zinc-induced lysosomal activation. Zinc under sub-lethal concentration contributes to lysosomal activation. Zinc promotes the function of V-ATPase by rapidly increasing the assembly of the V1 subunit and the V0 subunit on the lysosomal membrane. As a result, lysosomal acidification is improved, and the activity of proteolytic enzymes such as CTSB and CTSD is increased. Zinc also promotes the dephosphorylation of TFEB and its translocation to the nucleus. TFEB in the nucleus activates the expression of lysosomal degrading enzymes, including CTSB and CTSD, and the proteins constituting V-ATPase such as V0D1 and V1B2. Enhanced lysosomal acidification and biogenesis induce strong lysosomal activation, which can significantly contribute to the degradation of protein aggregates, especially when the autophagic flux is blocked.

Here, we show that zinc induced lysosomal biogenesis via TFEB activation and lysosomal acidification via increases in the assembly and expression of V-ATPase, which may ameliorate the accumulation of aggregated proteins in neurons of neurodegenerative diseases (Figure 8). Lysosomal acidification was induced immediately after exposure to zinc (Figure 5), which was followed by lysosomal biogenesis, including V-ATPase subunits and CTSB/CTSD (Figures 3, 6). Whereas a strong increase in the activity of CTSB and CTSD was observed from 1 h after zinc treatment (Figure 4), mild increases in the protein expression of CTSB and CTSD only in mature forms were observed in Western blot (Figure 3B). The change in activity was noticeable compared to the change in expression, possibly due to the simultaneous conversion of CTSB/CTSD into mature forms by concurrent lysosomal acidification. On the other hand, rapamycin failed to induce TFEB and CTSB/CTSD activation. Zinc but not rapamycin recovered autophagic flux arrested by lysosomal alkalization.

Zinc is an essential trace metal ion for living organisms. Especially in the brain, labile zinc is stored in synaptic vesicles at concentrations up to 1 mM or more (Frederickson et al., 2000) and plays a role in synaptic transmission. The level of labile zinc in the cytoplasm that was not bound to proteins is ∼100 pM (Hara et al., 2017), and neuronal death occurs when the zinc level exceeds ∼10 nM (Jiang et al., 2001). We confirmed that 25 μM zinc exposure to mouse primary cerebrocortical cultures used in the present study did not induce significant ROS generation and neuronal death (Supplementary Figure 1) and that the zinc concentration used for human H4 neuroglioma cells or human SK-N-BE(2)-C neuroblastoma cells also did not cause cell damage (data not shown). It is known that the concentration of zinc in the blood and cerebrospinal fluid (CSF) decreases with aging, which is accelerated in patients with neurodegenerative diseases including AD and PD (Molina et al., 1998; Du et al., 2017). Recently, it has been reported that zinc may be involved in autophagy and lysosomal function, and that zinc transporters such as ZnT2 and ZnT3 are necessary for lysosomal acidification (Rivera et al., 2018; Lee and Koh, 2021). We also showed that H_2_O_2_-induced or tamoxifen-induced AV formation and autophagy activation was mediated by zinc (Lee et al., 2009; Hwang et al., 2010). Liuzzi and Yoo observed that zinc depletion caused the significant suppression of autophagy and that the addition of zinc to medium stimulated autophagy in human hepatoma cells (Liuzzi and Yoo, 2013). Rudolf has also shown that, upon external exposure to zinc, it entered lysosomes and stimulated autophagy in human melanocytes (Rudolf and Rudolf, 2017). In addition, clioquinol, a zinc ionophore, was reported to activate autophagy and acidify lysosomal pH, resulting in the degradation of mutant huntingtin and amyloid-β oligomer (Park et al., 2011; Seo et al., 2015). However, it remains unknown how zinc controls autophagy or lysosomal function. Therefore, we attempted to elucidate the mechanism by which zinc regulates autophagy or lysosomes. First, we observed that a sub-lethal concentration of zinc rescued the arrest of autophagic flux by CQ or NH_4_Cl in mRGL-H4 glioma cells and primary cerebrocortical neuronal cultures. Zinc rapidly increased the protein expression and dephosphorylation levels of TFEB, and the translocation of TFEB from neurites to nucleus in mouse cerebrocortical neuronal cultures, leading to induction of the expression of CTSB and CTSD. We also found that the initial enhancement of protease activity of CTSB/CTSD was mainly due to the increase of V-ATPase assembly and resultant lysosomal acidification, and the sustained CTSB/CTSD activity was later maintained by the induction of their expression levels. Finally, we demonstrated that zinc reduced wild-type or mutant Tau-P301L accumulation in SK-N-BE(2)-C neuroblastoma cells.

In particular, we demonstrated zinc-induced TFEB activation and induction in this study. The subcellular localization and activity of TFEB are mainly regulated by its phosphorylation status. When two serine residues of TFEB, Ser142 and Ser211, are phosphorylated, TFEB is kept inactive in the cytosol. mTORC1 and extracellular signal-regulated kinase 2 (ERK2) are known to phosphorylate TFEB under nutrient-rich conditions in most cell types (Settembre et al., 2011, 2012; Martina et al., 2012; Roczniak-Ferguson et al., 2012), although protein kinase Cβ (PKCβ) phosphorylates the C-terminal region of TFEB upon stimulation with receptor activator of nuclear factor kB ligand (RANKL) in osteoclasts and then promotes lysosomal biogenesis (Ferron et al., 2013). Starvation or lysosomal stress releases mTORC1 from the lysosomal membrane and inactivates it, which induces the release of lysosomal Ca^2+^ through the Ca^2+^ channel mucolipin 1 (MCOLN1). Released calcium in turn activates the phosphatase calcineurin, leading to the dephosphorylation of TFEB. MCOLN1, also known as TRPML1 (transient receptor potential mucolipin 1), is a non-selective ion channel for Ca^2+^, Zn^2+^, Fe^2+^, and Mn^2+^, suggesting that TRPML1 may function in zinc-induced TFEB dephosphorylation and activation. Some reports have shown that the loss of TRPML1 function leads to the accumulation of free zinc in lysosomes and membranous vacuoles, induces lysosomal enlargement, and downregulates ZnT3 and ZnT4 expression levels, which results in intracellular zinc dyshomeostasis and lysosomal dysfunction. Similarly, growing evidence indicates that TRPML1 is important for the regulation of zinc homeostasis in relation to lysosomes, but no study shows that zinc acts directly on the expression or activity of TRPML1. In the present study, we examined whether the expression of TRPML1 was regulated by zinc and found that TRPML1 expression was not significantly affected by zinc (Figures 3A,B). We are planning to perform a further study to examine whether TRPML1 is involved in zinc-mediated TFEB dephosphorylation and translocation, and determine which molecules play critical roles in zinc-induced TFEB activation. In addition, TFEB activation increases its own transcription (Settembre et al., 2013), which further sustains lysosomal signaling. Here, we observed that exposure to a sub-lethal concentration of zinc simultaneously increased the protein level of TFEB as well as its dephosphorylation and translocation (Figures 3E,F). Although another study presented that zinc promoted the binding of metal response element-binding transcription factor (MTF)-1 to DNA at the promoter region of peroxisome proliferator-activated receptor (PPAR)α to induce transcriptional activation of the key genes related to autophagy and lipolysis (Wei et al., 2018), the present study is the first to show zinc-mediated activation and induction of TFEB. For further study, it is necessary to determine whether the expression levels of CTSB/CTSD and V-ATPase are mediated by zinc-induced TFEB activation and how zinc regulates TFEB dephosphorylation and activation.

V-ATPase is a proton pump that acidifies intracellular compartments in eukaryotic cells, and transports protons across the plasma membrane of certain specialized cells. V-ATPase is a large, multi-subunit complex composed of an integral V0 domain that translocates protons and a peripheral V1 domain that hydrolyzes ATP (McGuire et al., 2017). In the dissociated state, both the catalytic function of V1 and the proton transport function of V0 are inactive. Since so many essential processes are pH-dependent, the regulation of V-ATPase activity should be tightly controlled. Reversible dissociation of V1 domain and V0 domain is a major form of V-ATPase regulation and occurs in response to nutrient levels. In mammalian cells, amino acid starvation leads to the rapid and reversible assembly of V-ATPase on lysosome to enhance protein degradation, thereby increasing free amino acids (Stransky and Forgac, 2015). Glucose starvation also increases V-ATPase assembly and activity on lysosomes in mammalian cells (McGuire and Forgac, 2018). Since V-ATPase is an electrogenic proton pump, V-ATPase activity is also sensitive to the activity of other transporters. ZnT2 interacts with V-ATPase and the loss of ZnT2 disrupts V-ATPase assembly, resulting in impaired acidification of vesicles such as lysosomes and secretory vesicles in mammary glands (Rivera et al., 2018). Here, we show that zinc augmented the ATPase activity through a reversible induction of assembly and expression. First, we demonstrated that zinc rapidly promoted V-ATPase assembly and lysosomal acidification. Next, we found that the expression of V1B2 and V0D1 increased from 1 h after zinc treatment (Figure 6B), and that of V1A increased after 2 h zinc treatment (data not shown). These results are consistent with that of a previous study in which the expression of ATPase subunits was induced in a TFEB-dependent manner (Pena-Llopis et al., 2011). Future studies will focus on understanding the mechanism by which signaling pathways modulate zinc-mediated V-ATPase assembly, which subunit of the V-ATPase complex zinc directly binds to, or which ZnT2 activity is needed for zinc-induced V-ATPase assembly.

Rapamycin is a widely used inhibitor of mammalian target of rapamycin (mTOR). mTOR is a cytosolic Ser/Thr kinase belonging to the phosphatidylinositol kinase-related family of protein kinases and plays a key role in numerous cellular processes including autophagy, cell growth, and cell survival. mTOR forms two distinct complexes: mTOR complex 1 (mTORC1) and mTOR complex 2 (mTROC2). The main function of mTORC1 is the activation of anabolic processes including autophagy, whereas mTORC2 plays roles in regulating cell survival, proliferation, and shape (Oh and Jacinto, 2011; Rabanal-Ruiz et al., 2017; Szwed et al., 2021). Rapamycin binds to mTORC1, which prevents substrate recruitment at the active site, leading to the blocking of mTORC1 and a switch of cell metabolism toward a catabolic pathway. However, rapamycin cannot completely inhibit mTORC1. In particular, mTORC1-dependent protein synthesis is resistant to rapamycin. The phosphorylation of the translational inhibitor 4E-BP, a well-known substrate of mTORC1, was not inhibited by rapamycin (Thoreen et al., 2009), and the activity of TFEB, a master transcription factor of lysosomal biogenesis and substrate of mTORC1, was also resistant to rapamycin (Settembre et al., 2012). Here, we also observed that the subcellular localization of TFEB was not changed by rapamycin (Figures 3C,D), leading to no induction of CTSB and CTSD in mouse cerebrocortical neurons (Figure 4C). However, zinc notably induced the dephosphorylating activation and an increase in the expression levels of TFEB as well as TFEB translocation (Figures 2D–F). Furthermore, zinc rapidly activated V-ATPase assembly and then modified lysosomal acidification (Figures 5, 6). Based on this regulation by zinc, a block of autophagic flux by CQ or NH_4_Cl was significantly rescued by zinc but not by rapamycin (Figure 2). Finally, we observed that tau phosphorylation and accumulation were efficiently attenuated by zinc more markedly than by rapamycin in wild-type or mutant tau (tau-P301L)-overexpressing SK-N-BE(2)-C neuroblastoma cells (Figure 7). These results suggest that the activation of autophagosome formation by rapamycin may not be sufficient to restore protein degradation when autophagic flux is blocked. Our study suggested that developing a drug that can increase autophagic flux by modulating intracellular zinc homeostasis in neurons may be a good strategy for treating neurodegenerative diseases such as AD, PD, and HD.

## Data availability statement

The original contributions presented in this study are included in the article/Supplementary material, further inquiries can be directed to the corresponding authors.

## Ethics statement

All animal experimental procedures were reviewed and approved by the Animal Care and Use Committee of Sejong University and were conducted following the guidelines of the Care and Use of Laboratory Animals.

## Author contributions

Y-HK and JJH conceived the project and designed the experiments. K-RK, SEP, and J-YH performed the experiments. K-RK, SEP, JJH, and Y-HK wrote the manuscript. All authors analyzed and interpreted the results, read, and approved the final manuscript.

## Supplementary material

Supplementary Figure 1 Zinc-induced ROS generation and cell death. **(A)** Quantification graph of DCF fluorescence in mouse cerebrocortical cultures. Cultures were exposed to 25, 50, or 100 μM ZnCl_2_ or 100 μM H_2_O_2_ for 12 h. The mean intensity of DCF was quantified in a given microscopic field using Image J software (mean ± SEM, n = 9 different fields taken from ≥ 3 independent biological replicate experiments). **p* < 0.05, ^**^*p* < 0.01 or ^***^*p* < 0.001 by ANOVA with Dunnett’s test for *post hoc* analysis. **(B)** LDH release from dead cells 12 h after exposure to the indicated concentration of ZnCl_2_ in mouse cerebrocortical culture (mean ± SEM, *n* = 8 cultures), ^****^*p* < 0.0001 by ANOVA.
