# Supplementary figures and images for: Zinc enhances autophagic flux and lysosomal function through transcription factor EB activation and V-ATPase assembly
Source: Front Cell Neurosci. 2022 Sep 29;16:895750. doi: 10.3389/fncel.2022.895750 (PMC9558701; doi:10.3389/fncel.2022.895750)

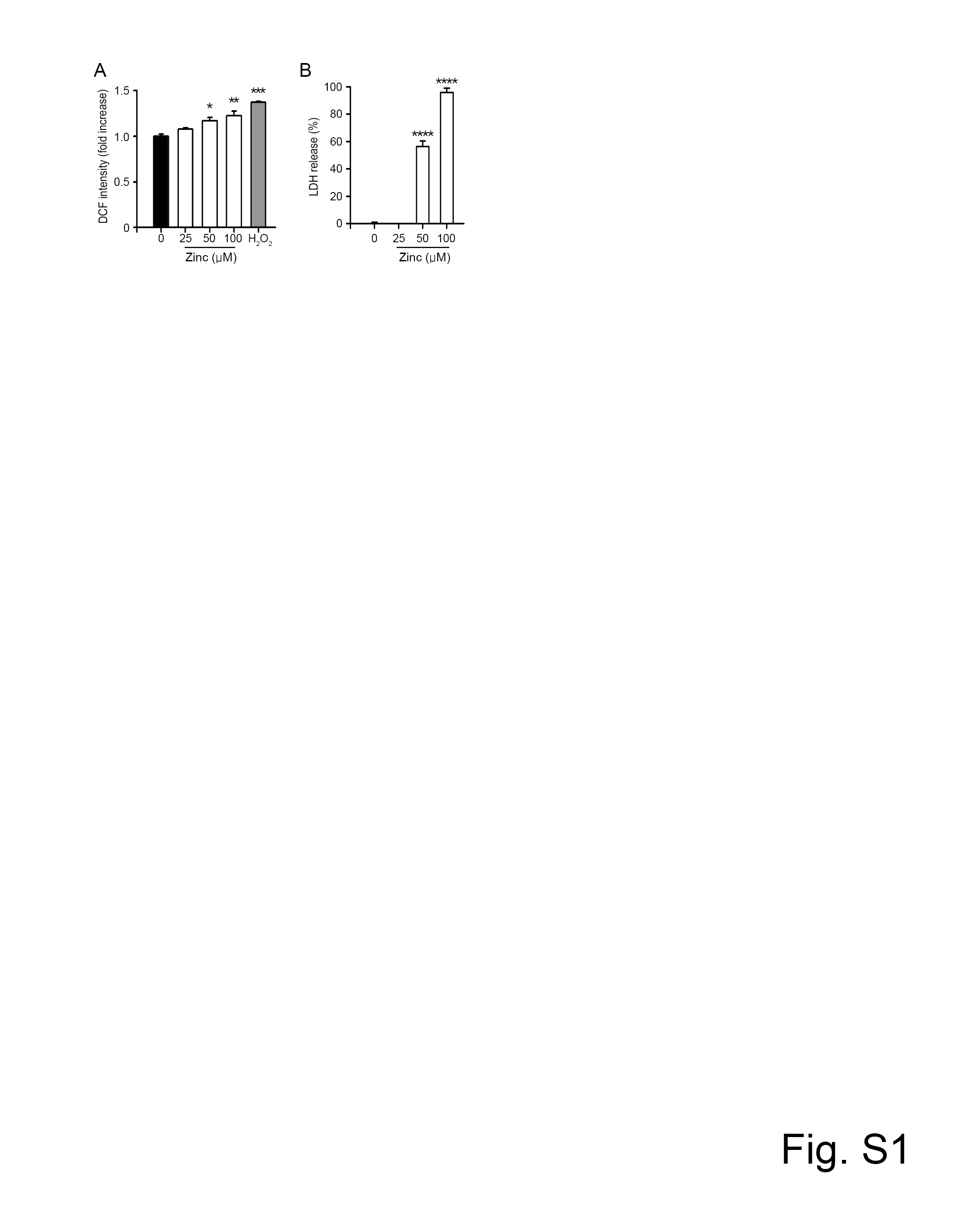

Supplement: Supplementary Figure 1 — Zinc-induced ROS generation and cell death. (A) Quantification graph of DCF fluorescence in mouse cerebrocortical cultures. Cultures were exposed to 25, 50, or 100 μM ZnCl2 or 100 μM H2O2 for 12 h. The mean intensity of DCF was quantified in a given microscopic field using Image J software (mean ± SEM, n = 9 different fields taken from ≥ 3 independent biological replicate experiments). *p < 0.05, **p < 0.01 or ***p < 0.001 by ANOVA with Dunnett’s test for post hoc analysis. (B) LDH release from dead cells 12 h after exposure to the indicated concentration of ZnCl2 in mouse cerebrocortical culture (mean ± SEM, n = 8 cultures), ****p < 0.0001 by ANOVA. [file Image_1.tif]
